# STYK1/NOK Promotes Metastasis and Epithelial-Mesenchymal Transition in Non-small Cell Lung Cancer by Suppressing FoxO1 Signaling

**DOI:** 10.3389/fcell.2021.621147

**Published:** 2021-07-06

**Authors:** Yuanyang Lai, Fang Lin, Xuejiao Wang, Jiao Zhang, Jinghua Xia, Ying Sun, Miaomiao Wen, Xiaofei Li, Zhipei Zhang, Jinbo Zhao

**Affiliations:** ^1^Department of Thoracic Surgery, Tangdu Hospital, The Air Force Medical University, Xi’an, China; ^2^Department of Clinical Diagnosis, Tangdu Hospital, The Air Force Medical University, Xi’an, China

**Keywords:** STYK1(NOK), NSCLC, metastasis, EMT, FoxO1

## Abstract

**Aims:**

Serine/threonine/tyrosine kinase 1 (STYK1) has been previously shown to have oncogenic properties, and emerging evidence suggests that STYK1 expression correlates with epithelial-mesenchymal transition (EMT). However, the mechanism of STYK1 involvement in oncogenesis remains unknown. The present study aimed to elucidate how STYK1 expression level relates to the metastasis, migration, invasion, and EMT in non-small cell lung cancer (NSCLC) and to determine the molecular mechanism of STYK1 effects.

**Methods:**

Serine/threonine/tyrosine kinase 1 (STYK1) expression level and its relationship with the prognosis of NSCLC were determined using the ONCOMINE database and clinical cases. Non-small cell lung cancer cell lines with the overexpression or knockdown of STYK1 were established to determine whether STYK1 promotes cell migration, invasion, and EMT *in vitro* and *in vivo*. In addition, a constitutively active FoxO1 mutant (FoxO1AAA) was used to examine the role of FoxO1 in the STYK1-mediated upregulation of metastasis and EMT in NSCLC.

**Results:**

Serine/threonine/tyrosine kinase 1 (STYK1) was upregulated in NSCLC tissues and cell lines, and its overexpression correlated with poor prognosis in patients with NSCLC after surgery. Enhanced expression of STYK1 potentiated the migration, invasion, and EMT in SW900 cells, thereby promoting metastasis, whereas knockdown of STYK1 inhibited these cellular phenomena in Calu-1 cells. Furthermore, STYK1 expression was positively related to the level of phosphorylated-FoxO1, whereas the constitutively active FoxO1 mutant protected against the positive effect of STYK1 overexpression on cell migration, invasion, and EMT.

**Conclusion:**

Serine/threonine/tyrosine kinase 1 (STYK1) was upregulated in NSCLC and correlated with poor clinical outcomes. In addition, STYK1 suppressed FoxO1 functions, thereby promoting metastasis and EMT in NSCLC.

## Introduction

Metastasis is one of the most important factors contributing to the poor prognosis of lung cancer. Approximately 57% of patients with lung cancer have metastatic lesions at initial diagnosis, and their 5-year survival rate is only 5.8%, which is far lower than that of patients with localized lung cancer (SEER, 2020; Siegel et al., 2020). Better control of metastasis could greatly improve the prognosis of lung cancer.

According to the *invasion-metastasis cascade* theory, epithelial-mesenchymal transition (EMT) plays an important role in cancer metastasis (Talmadge and Fidler, 2010; Hanahan and Weinberg, 2011; Yeung and Yang, 2017). Epithelial-mesenchymal transition, in particular type 3 EMT, facilitates the dissociation, migration, and invasion of epithelial cancer cells by promoting changes in cell adhesion, morphology, mobility, resistance to anoikis, and extracellular matrix degradation, thereby participating in the initiation and maintenance of metastasis. This is directly evidenced by the fact that in patients with progressive metastasis, circulating tumor cells frequently express epithelial and mesenchymal markers simultaneously, whereas primary tumor cells rarely co-express epithelial and mesenchymal proteins (Armstrong et al., 2011; Yu et al., 2013; Wu et al., 2015). Therefore, it is rational to focus on EMT in order to investigate the mechanisms of metastasis.

Expression of STYK1 (serine/threonine/tyrosine kinase 1, also known as NOK, a novel oncogene with kinase-domain) is aberrant in many malignancies (Moriai et al., 2006; Jackson et al., 2009; Kondoh et al., 2009; Orang et al., 2014; Chen et al., 2016, 2017; Hu et al., 2018). Moreover, overexpressed STYK1 likely stimulates cancer development by sustaining proliferative signaling, leading to abnormal proliferation of cancer cells (Chung et al., 2009; Cao et al., 2016; Chen et al., 2016), enhancing the resistance of tumor cells to programmed cell death (Lai et al., 2019; Shi et al., 2019), promoting the Warburg effect, remodeling cellular energetics in malignant cells (Shi et al., 2017; Zhao et al., 2017), and stimulating angiogenesis and lymphangiogenesis during tumor progression (Liu et al., 2016). In addition, a high level of STYK1 downregulates the expression of E-cadherin (Chen et al., 2005, 2017) and induces EMT via MAPK/ERK and PI3K/AKT signaling (Chen et al., 2016, 2017; Wang et al., 2016), indicating that STYK1 expression correlates with EMT and metastasis, although the precise mechanisms underpinning this relationship need to be further studied.

The forkhead box O (FoxO) family of proteins are shared downstream molecules of the MAPK/ERK and PI3K/AKT pathways (KEGG, 2020), and they could also serve as effectors after STYK1 activation. FoxO1, a representative protein of this family, functions as a *super transcription factor* and regulates genes involved in the proliferation, apoptosis, oxidative stress, and metabolic modulation. Phosphorylation deactivates FoxO1 and prompts its dissociation from DNA and translocation from the nucleus to the cytoplasm, subsequently leading to the genesis of neoplasms and other diseases (Jiang et al., 2018; Xing et al., 2018). FoxO1 reverses the development of EMT by inhibiting EMT transcription factors such as Twist (Wu et al., 2014) and ZEB2 (Dong et al., 2017). The above-mentioned studies indicated that targeting FoxO1 could be a reasonable strategy to inhibit EMT induction by STYK1. However, details about the interactions in the STYK1-FoxO1-EMT axis remain unknown.

In the present study, we examined the relationship between STYK1 expression and survival in patients with NSCLC using bioinformatics and analysis of surgical samples. In addition, NSCLC cell lines with overexpression or knockdown of STYK1 were constructed to determine whether and how STYK1 affected cell migration, invasion, and EMT *in vitro* and *in vivo*. Furthermore, a constitutively active FoxO1 mutant (FoxO1AAA; mutant phosphorylation sites: T24A, S256A, and S319A) was utilized to determine the role of FoxO1 in the effects of STYK1 on metastasis and EMT in NSCLC. Our results provide new insights into the oncogenicity of STYK1 and reveal new approaches to control metastasis in NSCLC.

## Materials and Methods

### Patients and Sample Collection

Tumor specimens and adjacent normal tissues from 208 NSCLC patients who underwent surgery without neoadjuvant therapy at our Institute between January 2011 and December 2014 were collected and cryopreserved immediately after resection. The complete 5-year follow-up and other clinicopathological data were archived from our Tumor Specimen Database. Tumor tissues from another 70 NSCLC patients who underwent surgery without neoadjuvant therapy between July 2017 and June 2018 were also collected for tissue microarray analysis. Written consent for studies of the samples was obtained from all patients. The study was approved by the Institutional Ethics Review Board of Tangdu Hospital.

### Cells and Mice

Human bronchial epithelial cells (HBE) and human lung cancer cell lines (SPC-A-1, Sk-lu-1, Calu-1, A549, H520, and SW900) were preserved in our laboratory. The cells were cultured routinely as previously described (Lai et al., 2019).

Four- to six-week-old male BABL/c nude mice were obtained and maintained at the Experimental Animal Center of the Air Force Medical University (Xi’an, China). The animal experiments were performed in accordance with the *Guide for the Care and Use of Laboratory Animals* (National Research Council Committee for the Update of the Guide for the Care and Use of Laboratory, 2011) and were approved by the Laboratory Animal Welfare and Ethics Committee of the Air Force Medical University.

### Bioinformatic Analysis

The ONCOMINE database^1^ was used to predict the expression of STYK1 and its relationship with prognosis in NSCLC, as previously described (Lai et al., 2019). In brief, filters “Gene: STYK1,” “Analysis Type: Lung Cancer vs. Normal Analysis,” “Sample Type: Surgical Specimen,” “Data Type: mRNA” were used to mine studies for the meta-analysis and differential analysis of STYK1 expression, whereas filters such as “Gene: STYK1,” “Cancer Type: Lung Cancer,” “Sample Type: Surgical Specimen,” “Data Type: mRNA,” “Clinical Outcome: Survival Status” were applied to obtain datasets for the survival analysis. After filtering, datasets using the same reporters were included. Information about expression value, tissue type, legend value, sample name, overall survival status, and overall follow-up time was extracted from the included datasets. For the survival analysis, the median of expression value was used to distinguish between high and low levels of *STYK1* mRNA.

### Immunohistochemistry (IHC)

All samples were embedded in paraffin following paraformaldehyde fixation, ethanol gradient dehydration, and xylene treatment to make tissues transparent. Paraffin-embedded tumor specimens and corresponding normal tissues from the 208 cases were cut into sections (3 μm thick), and paraffin-embedded tumor specimens from another 70 patients were constructed for tissue microarray. Immunohistochemistry was performed on paraffin sections and tissue microarray as previously described (Xiong et al., 2017), and the histochemical score (h-score) was introduced for the semiquantitative analysis (Budwit-Novotny et al., 1986). The h-score of STYK1 that was higher or lower than the median was defined as high or low expression of STYK1 for data analysis, respectively. Rabbit anti-human polyclonal antibodies against STYK1 (1:80; Cat.: ab97451, Abcam, Cambridge, MA, United States) and phosphorylated (p)-FoxO1 (1:60; Cat.: ab131339, Abcam) were used as primary antibodies.

### Total RNA Extraction and RT-qPCR

Total RNA was isolated from the cells and reverse transcribed into cDNA using an RNAprep Pure Cell/Bacteria Kit (Cat.: DP430; TIANGEN BIOTECH, Beijing, China) and a Thermo Scientific RevertAid First Strand cDNA Synthesis Kit (Cat.: K1622; Thermo Fisher Scientific, Waltham, MA, United States), respectively, as previously described (Lai et al., 2019). RT-qPCR was performed using UltraSYBR Mixture (Cat.: CW0957; CWBIO, Beijing, China) and an Agilent Technologies Stratagene Mx3005P real-time PCR system. The profile of 40 thermal cycles was as follows: denaturation for 40 s at 95°C, annealing for 30 s at 61°C, and extension for 45 s at 72°C. The results were normalized to the level of β-actin mRNA, and the relative mRNA levels of target genes were quantified using the 2^–ΔΔCt^ method. The primer sequences were as follows: STYK1, forward (5′-TCGAGCCAATATGAACACTGGG-3′) and reverse (5′-TCGCCCTAAGAAATCTTGTACCT-3′); FoxO1, forward (5′-TCGTCATAATCTGTCCCTACACA-3′) and reverse (5′-CGGCTTCGGCTCTTAGCAAA-3′); β-actin, forward (5′-CTCCATCCTGGCCTCGCTGT-3′) and reverse (5′-GCTGTCACCTTCACCGTTCC-3′).

### Western Blot Analysis

STYK1, Snail, ZEB2, E-cadherin, vimentin, p-FoxO1, and total FoxO1 protein levels were determined by western blotting using previously described protocols (Lai et al., 2019). The following primary antibodies were used: rabbit polyclonal anti-human STYK1 (1:1,000; Cat.: ab97451, Abcam), rabbit monoclonal anti-human Snail (1:1,000; Cat.: #3879S, CST, Danvers, MA, United States), rabbit polyclonal anti-human ZEB2 (1:1,000; Cat.: ab138222, Abcam), rabbit monoclonal anti-human E-cadherin (1:10,000; Cat.: ab40772, Abcam), rabbit monoclonal anti-human vimentin (1:1,000; Cat.: ab92547, Abcam), rabbit polyclonal anti-human p-FoxO1 (1:500; Cat.: ab131339, Abcam), and rabbit monoclonal anti-human FoxO1 (1:1,000; Cat.: ab52857, Abcam). Rabbit polyclonal anti-human β-actin (1:1,000; Cat.: ab8227, Abcam) or rabbit monoclonal anti-human GAPDH (1:1,000; Cat.: #5174S, CST) were used as internal controls.

### Cell Transfection

Lentiviruses encoding STYK1 and FoxO1AAA, short hairpin RNA plasmids targeting *STYK1*, and corresponding controls were constructed by GeneChem (Shanghai, China). FoxO1AAA is a constitutively active FoxO1 mutant (mutant phosphorylation sites: T24A, S256A, and S319A). Lentiviral expression of FoxO1AAA was used to enhance FoxO1 activities in cell lines. Plasmids carrying luciferase were kindly gifted by the Department of Biochemistry, School of Basic Medicine of the Air Force Medical University (Xi’an, China). Transduction of SW900 cells with lentiviruses encoding STYK1 and FoxO1AAA was performed as previously described (Lai et al., 2019).

Plasmid transfection was done by electroporation using an ECM 830 Electroporation System (BTX, Holliston, MA, United States) according to the manufacturer’s instructions. Briefly, after being trypsinized, washed, and resuspended in serum-free RPMI-1640 medium (Cat.: SH30809.01B; GE Life Sciences, Pittsburgh, PA, United States), the cells (10^6^/sample) were pipetted into disposable cuvettes (Cat.: 45-0126; BTX, Holliston, MA, United States) and gently mixed with 40 μg of plasmids. The cell suspension was then electroporated with the following electrical characteristics: voltage, 180 V; pulse length, 40 ms; pulse interval, 10 s; and multiple pulsing, 2 pulses. In 72 h after transfection, stable cell colonies were selected using the RPMI-1640 medium supplemented with 10% fetal bovine serum (Cat.: 900-108; GEMINI Bio-Products, Woodland, CA, United States) and 2 μg/mL puromycin (Cat.: 60210ES25; YEASEN, Shanghai, China), expanded, and maintained in the RPMI-1640 medium with 10% FBS and 1 μg/mL puromycin.

### Determination of Cell Migration and Invasion

Cellular migration and invasion capacities were assessed using a combination of real-time cell analysis (RTCA) and crystal violet staining. Real-time cell analysis was performed using an xCELLigence RTCA DP instrument (ACEA Biosciences) following the manufacturer’s instructions. For the cell migration assay, after the background impedance measurement of the CIM-16 plate (a 16-well electronically integrated Boyden chamber), cells were suspended in the serum-free RPMI-1640 medium and added to the upper chamber of the CIM-16 plate (40,000 cells/well). The CIM-16 plate was then loaded into the xCELLigence RTCA DP instrument and incubated in a humidified incubator at 37°C in the atmosphere of 95% air and 5% CO_2_. Cell migration was scanned every 15 min for 48 h, and the cell index was measured and corrected using the background impedance for later analyses.

For the cell invasion assay, the above steps of the cell migration assay were carried out immediately after Matrigel coating of the upper chamber of the CIM-16 plate.

At the endpoint of the scanning, the upper chamber of the CIM-16 plate was disassembled, and the migrated or invaded cells on the CIM membrane were stained with crystal violet solution and visualized using an upright microscope (AXIOSKOP 40, Carl Zeiss AG, Jena, Germany) according to the standard protocol.

### Xenograft Metastatic NSCLC Model

Wild-type and STYK1 overexpressing SW900 cells were transfected with plasmids carrying luciferase, and injected into the tail vein of BALB/c mice to generate a xenograft metastatic NSCLC model (*n* = 5; 5 × 10^6^ cells/mice). In 4–5 weeks after tail vein injection of tumor cells, the mice were intraperitoneally administered D-luciferin potassium salt (150 μg/g body weight; Cat.: ab143655, Abcam) for *in vivo* bioluminescence imaging to monitor metastatic sites using a Caliper IVIS Lumina II multispectral imaging system. Qualitative imaging and quantification of photons emitted by the metastases (radiance; p/sec/cm^2^/sr) were performed using the Living Image Software. Mice were then sacrificed and their lungs were dissected, paraffin-embedded, sectioned, and stained with hematoxylin/eosin to confirm the presence of metastases. The metastatic foci were counted in 5 randomly selected fields in each section at 10 X magnification, and 10 sections per mouse were analyzed.

### Statistical Analysis

The data are presented as the median with interquartile range or as the means ± standard error of the mean of triplicate experiments. Statistical calculations were performed using GraphPad Prism 8 software (GraphPad Inc., San Diego, CA, United States). The Pearson’s χ^2^ test was used to analyze the relationships between STYK1 expression and clinicopathological features. The COX regression analysis was used to define prognostic factors. Group differences were determined using non-parametric or parametric tests according to the normality test. Survival analysis was assessed using Kaplan-Meier curves and the log-rank test. Non-parametric Spearman correlation was used for the analysis of correlation between STYK1 and p-FoxO1 expression levels. Effects were considered statistically significant if *P* < 0.05.

## Results

### Upregulation of STYK1 Expression in NSCLC Tissues and Cells and Correlation of STYK1 Expression Level With Poorer Prognosis

**FIGURE 1:**
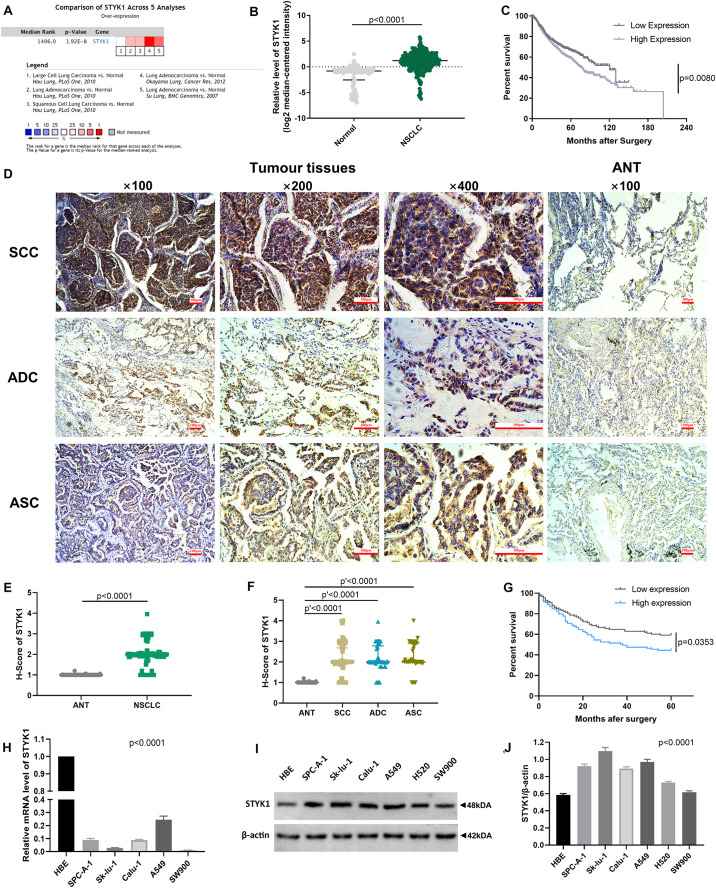
STYK1 expression is upregulated in NSCLC tissues and cells and correlates with poor prognosis. **(A)** *STYK1* mRNA level evaluated by meta-analysis; data were mined from three ONCOMINE datasets (Su et al., 2007; Hou et al., 2010; Okayama et al., 2012). **(B)** *STYK1* mRNA levels in NSCLC (*n* = 345) and normal lung tissues (*n* = 115) from previous ONCOMINE datasets; *P* < 0.0001, Mann-Whitney test. **(C)** Relationship between *STYK1* mRNA level and overall survival in 938 NSCLC cases from five ONCOMINE datasets (Raponi et al., 2006; Shedden et al., 2008; Kuner et al., 2009; Hou et al., 2010; Okayama et al., 2012) (high/low expression was defined as mRNA expression value higher/lower than the median); *P* = 0.0080, log-rank Mantel-Cox test. **(D)** Typical STYK1 IHC signal in NSCLC and adjacent normal lung tissues. **(E,F)** H-scores of STYK1 in NSCLC and adjacent normal lung tissues; *P* < 0.0001, Wilcoxon matched-pairs signed rank; *P*′ < 0.0001, Kruskal-Wallis test followed by Dunn’s multiple comparisons test. **(G)** Comparison of post-operative survival curves between patients with high or low STYK1 expression (*n* = 208; high or low expression, h-score higher or lower than the median); *P* = 0.0353, log-rank Mantel-Cox test. **(H–J)** mRNA, immunoblots, and semiquantitative analysis of STYK1 expression in lung cancer cell lines; *P* < 0.0001, ANOVA. SCC, squamous cell carcinoma; ADC, adenocarcinoma; ASC, adenosquamous carcinoma; ANT, adjacent normal lung tissues.

We first used datasets from the public ONCOMINE database to evaluate STYK1 expression and its relationship with prognosis in NSCLC. A total of 345 NSCLC samples and 115 normal lung tissue samples from three datasets (Su et al., 2007; Hou et al., 2010; Okayama et al., 2012) and 938 NSCLC cases from five datasets (Raponi et al., 2006; Shedden et al., 2008; Kuner et al., 2009; Hou et al., 2010; Okayama et al., 2012) were used for the meta-analysis, differential analysis (Figures 1A,B), and survival analysis (Figure 1C), respectively. We found that STYK1 expression was upregulated in NSCLC tissues compared to that in normal lung tissues, and that patients with high STYK1 expression had shorter overall survival.

Next, IHC (Figure 1D) and semiquantitative analysis of 208 pairs of resected NSCLC and adjacent normal tissues confirmed the upregulation of STYK1 in three common types of NSCLC (Figures 1E,F). The Pearson χ^2^ test showed that high STYK1 expression correlated with G3–G4, T3–T4, N2–N3, and III–IV tumors and with the number of deaths in 5 years after surgery (Table 1). In addition, the survival analysis demonstrated that patients with a high level of STYK1 expression had a lower 5-year survival rate after surgery (Figure 1G). Additional univariate Cox regression analysis demonstrated that high STYK1 expression, T, N, and AJCC 8th stage were significant prognostic factors after surgery for patients with NSCLC; however, follow-up multivariate analysis failed to identify those factors as independent prognostic factors (Table 2).

**TABLE 1:** Relationship between clinicopathological features and STYK1 expression in patients with NSCLC.

**Clinicopathological parameters**	**STYK1 expression**		**Pearson χ^2^**	***P***
	**Low (n,%)**	**High (n,%)**		
**Gender**				
Male	82, 51.3	78, 48.8	0.370	0.543
Female	27, 56.3	21, 43.8		
**Age**				
>61	51, 50.5	50, 49.5	0.287	0.592
≤ 61	58, 54.2	49, 45.8		
**Smoking**				
Never	37, 55.2	30, 44.8	0.315	0.575
Ever	72, 51.1	69, 48.9		
**Histology**				
SCC	60, 54.5	50, 45.5	1.954	0.377
ADC	32, 55.2	26, 44.8		
ASC	17, 42.5	23, 57.5		
**Differentiation**				
G1–G2	29, 67.4	14, 32.6	4.915	0.027*
G3–G4	80, 48.5	85, 51.5		
**T stage**				
T1–2	70, 59.3	48, 40.7	5.233	0.022*
T3–4	39, 42.7	51, 57.3		
**N stage**				
N0–1	91, 64.5	50, 35.5	25.844	<0.001*
N2–3	18, 26.9	49, 73.1		
**AJCC 8^*th*^ stage**				
I-II	80, 72.7	30, 27.3	38.662	<0.001*
III-IV	29, 29.6	69, 69.7		
**Survival state**				
Alive	64, 59.3	44, 40.7	4.233	0.040*
Dead	45, 45.0	55, 55.0		

*P < 0.05; low or high expression, h-score lower or higher than the median; SCC, squamous cell carcinoma; ADC, adenocarcinoma; ASC, adenosquamous carcinoma.

**TABLE 2:** Univariate and multivariate Cox regression analysis of prognostic factors for 5-year postoperative survival in patients with NSCLC.

	**Univariate**			**Multivariate**		
	***P***	**HR**	**95% CI**	***P***	**HR**	**95% CI**
STYK1 expression (High vs. low)	0.049*	1.487	1.002–2.206	0.410	1.202	0.776–1.861
Differentiation (G3–G4 vs. G1–G2)	0.161	1.247	0.916–1.698			
T (T3–4 vs. T1–2)	0.011*	1.664	1.121–2.469	0.127	1.452	0.900–2.344
N (N2–3 vs. N0–1)	0.027*	1.576	1.054–2.356	0.578	1.186	0.651–2.161
AJCC 8^*th*^ stage (III–IV vs. I–II)	0.005*	1.760	1.184–2.617	0.598	1.198	0.612–2.347

*P < 0.05; low or high expression, h-score lower or higher than the median.

We detected STYK1 expression in NSCLC cell lines and HBE cells using RT-qPCR and western blotting. STYK1 protein was upregulated in NSCLC cells compared to its expression in HBE cells (Figures 1I,J), whereas *STYK1* mRNA level was surprisingly much lower in tumor cells (Figure 1H). SW900 and Calu-1 Cell lines were used for further gene manipulations.

### Induction of Migration, Invasion, and EMT of NSCLC Cells by STYK1 *in vitro*

**FIGURE 2:**
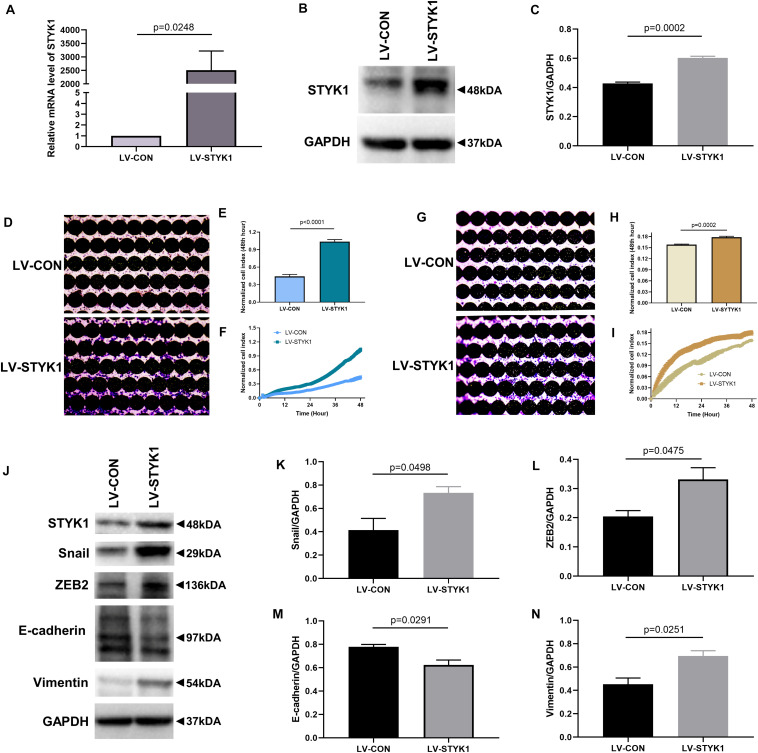
STYK1 overexpression upregulates migration, invasion, and EMT in SW900 cells. **(A–C)** (Lai et al., 2019) STYK1 expression in SW900 cells after transduction with STYK1 encoding lentiviruses; *P* = 0.0248 and *P* = 0.0002, Student’s *t*-test. **(D)** Capacity for cell migration after transduction with lentiviruses encoding STYK1; cells were stained with crystal violet at the endpoint of RTCA. **(E)** Normalized cell index after lentiviral STYK1 overexpression at the endpoint of RTCA; *P* < 0.0001, Student’s *t*-test. **(F)** Cell index curves after lentiviral STYK1 overexpression. **(G–I)** Cell invasion capacity after lentiviral STYK1 overexpression evaluated by RTCA and crystal violet staining; *P* = 0.0002, Student’s *t*-test. **(J–N)** Typical immunoblots and semiquantitative analysis of EMT biomarkers after transduction with lentiviruses encoding STYK1; *P* = 0.0498, *P* = 0.0475, *P* = 0.0291, and *P* = 0.0251, Student’s *t*-test. LV-CON, control SW900 cells; LV-STYK1, SW900 cells with lentiviral STYK1 overexpression.

To investigate whether STYK1 induced migration, invasion, and EMT, we first used lentiviruses to augment STYK1 expression in SW900 cells. Significant overexpression of STYK1 was observed by RT-qPCR and immunoblotting in SW900 cells after their transduction with lentiviruses carrying *STYK1* (Figures 2A–C) (Lai et al., 2019). Enhanced cell migration and invasion capacities were also observed after lentiviral transduction using the RTCA assay and crystal violet staining (Figures 2D–I). In addition, the results of immunoblots and semiquantitative analysis showed that overexpression of STYK1 in SW900 cells promoted EMT, as was evidenced by increases in the expression of Snail, ZEB2, and vimentin, and a decrease in E-cadherin expression (Figures 2J–N).

**FIGURE 3:**
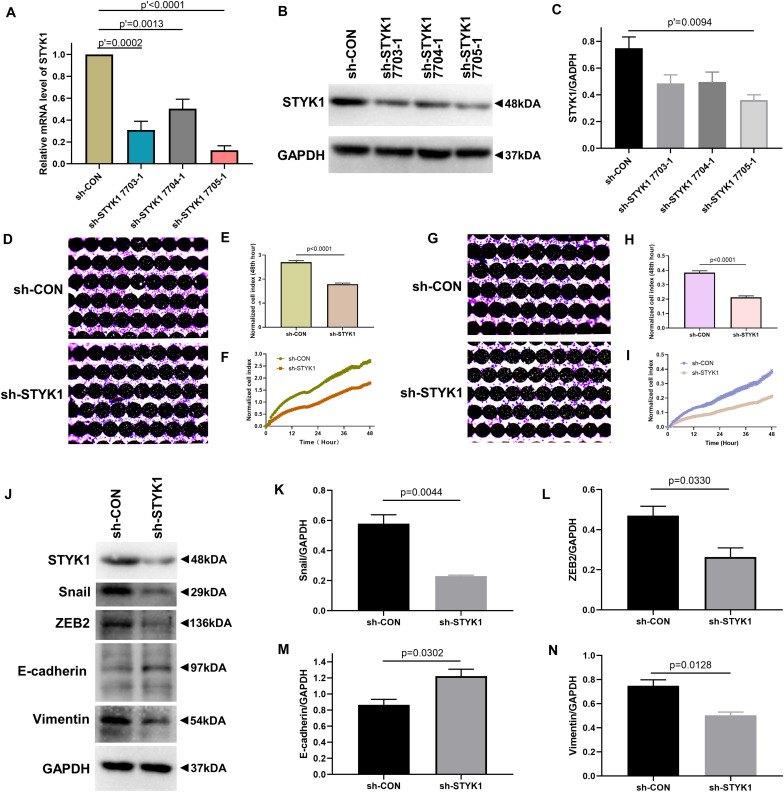
Knockdown of STYK1 suppresses migration, invasion, and EMT in Calu-1 cells. **(A–C)** Transfection efficiency of *STYK1*-targeting shRNAs in Calu-1 cells; sh-STYK1 7705-1 was adopted for further knockdown experiments. *P*′ < 0.0001, *P*′ = 0.0013, *P*′ = 0.0002 and *P*′ = 0.0094, ANOVA followed by the Dunnett’s multiple comparisons test. **(D–F)** Migration capacity of Calu-1 cells after shRNA transfection, evaluated by RTCA and crystal violet staining; *P* < 0.0001, Student’s *t*-test. **(G–I)** Invasion potential of Calu-1 cells after shRNA transfection; *P* < 0.0001, Student’s *t*-test. **(J–N)** Representative immunoblots and semiquantitative analysis of EMT biomarkers after shRNA transfection; *P* = 0.0044, *P* = 0.0330, *P* = 0.0302, and *P* = 0.0128, Student’s *t*-test. sh-CON, control Calu-1 cells; sh-STYK1, Calu-1 cells transfected with shRNA plasmids targeting *STYK1*.

We further evaluated the effects of STYK1 knockdown in Calu-1 cells on cell migration, invasion, and EMT. shRNA plasmids targeting STYK1 were electroporated into the cells. shRNA 7705-1 was confirmed to be the most effective plasmid to silence STYK1 expression, so it was used for the subsequent RTCA assay and western blotting (Figures 3A–C). As expected, knockdown of STYK1 inhibited the capacities of Calu-1 cells to migrate, invade, and undergo EMT (Figures 3D–N).

Taken together, these results indicate that STYK1 expression positively affected the migration, invasion, and EMT of NSCLC cells *in vitro.*

### Stimulation of Metastasis of NSCLC Cells by STYK1 *in vivo*

**FIGURE 4:**
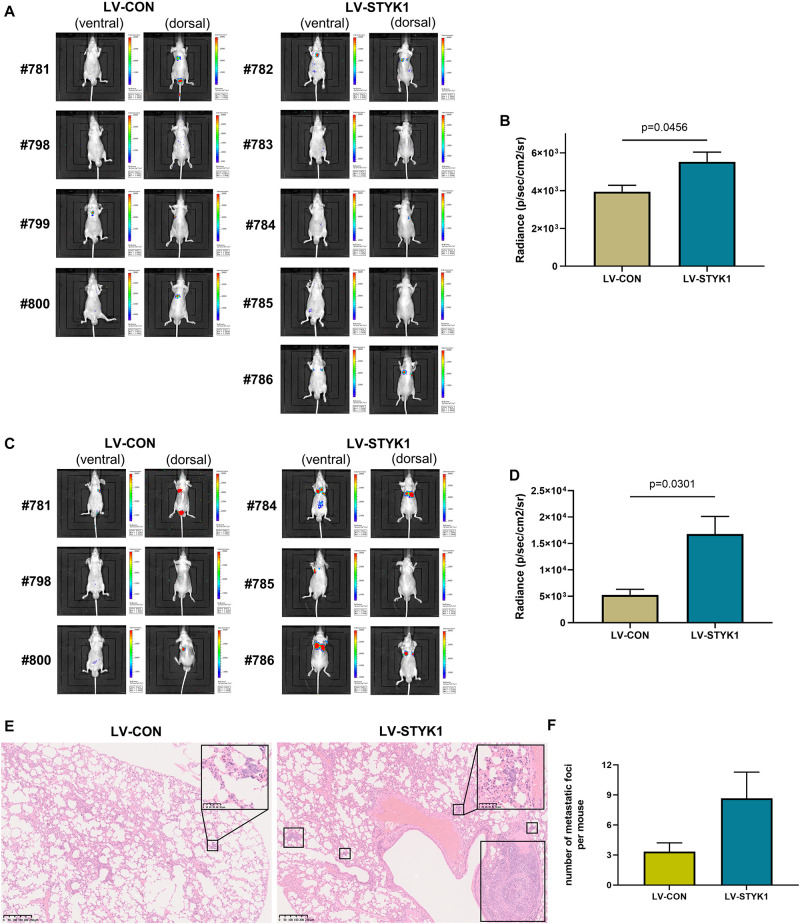
Overexpression of STYK1 promotes metastasis of SW900 cells in nude mice. **(A)** Luminescent images of nude mice in 4 weeks after tail vein injection of SW900 cells. **(B)** Quantitative analysis of radiance in 4 weeks after tail vein injection of SW900 cells; *P* = 0.0456, Student’s *t*-test. **(C,D)** Luminescent images of nude mice and quantification of radiance in 5 weeks after tail vein injection of SW900 cells; *P* = 0.0301, Student’s *t*-test. **(E)** Representative histological sections of lung metastatic lesions. **(F)** Comparison of the number of lung metastasis between groups (three mice per group); *P* = 0.1243, Student’s *t*-test. LV-CON, mice injected with control SW900 cells; LV-STYK1, mice injected with SW900 cells STYK1 overexpressing.

To assess the effect of STYK1 on the metastasis of NSCLC cells *in vivo*, wild-type and STYK1 overexpressing SW900 cells were transfected with plasmids carrying luciferase and injected via the tail vein into nude mice to construct a xenograft metastatic NSCLC model. The results of *in vivo* imaging revealed that 4 weeks after tail vein injection of tumor cells, mice harboring STYK1 overexpressing SW900 cells had more metastatic sites (Figure 4A) and emitted more photons than mice injected with wild-type cells (Figure 4B). Such differences were much more significant on week 5 after tumor cell injection (Figures 4C,D). Furthermore, hematoxylin/eosin staining showed that lung metastatic foci in mice injected with STYK1 overexpressing SW900 cells outnumbered those in mice that received wild-type SW900 cells (Figures 4E,F). These results demonstrate that the upregulation of STYK1 expression in NSCLC cells promoted metastasis *in vivo*.

### Activation of Migration, Invasion, and EMT by STYK1 in NSCLC Cells *via* FoxO1 Repression

**FIGURE 5:**
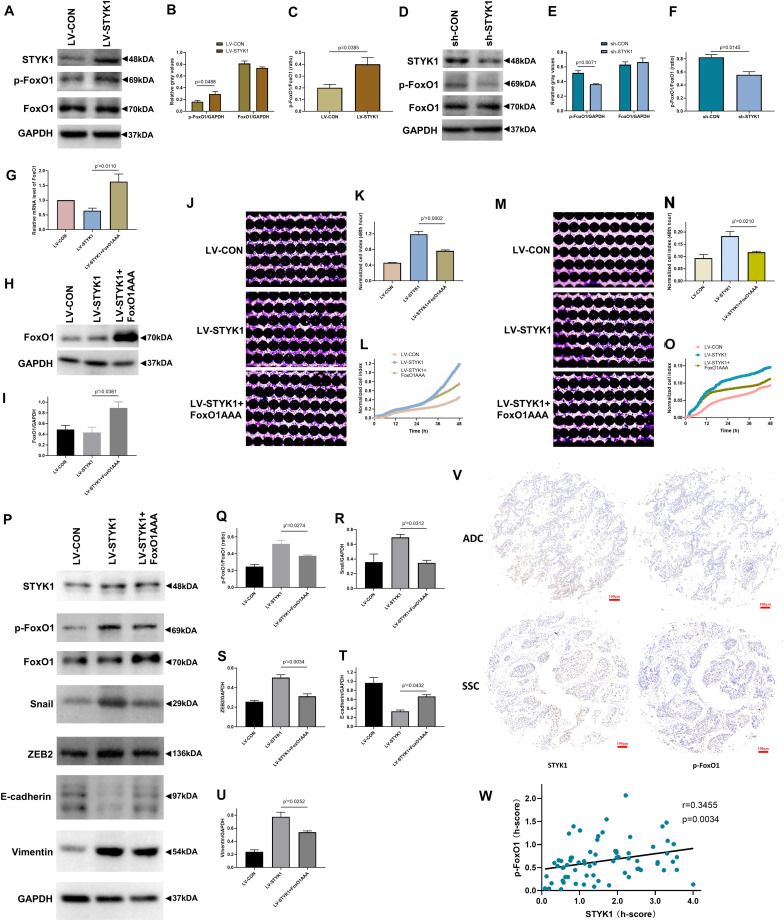
STYK1 activates migration, invasion, and EMT in NSCLC cells by repressing FoxO1. **(A–C)** Immunoblots and semiquantitative analysis of p-FoxO1 and FoxO1 levels, as well as the p-FoxO1/FoxO1 ratio after lentiviral STYK1 overexpression; *P* = 0.0488 and *P* = 0.0385, Student’s *t*-test. **(D–F)** Changes in p-FoxO1 and FoxO1 levels, as well as in the p-FoxO1/FoxO1 ratio after transfection with shRNA targeting *STYK1*; *P* = 0.0071 and *P* = 0.0145, Student’s *t*-test. **(G–I)** Confirmation of lentiviral expression of FoxO1AAA by RT-qPCR and western blot; *P*′ = 0.0110 and *P*′ = 0.0381, ANOVA followed by the Tukey’s multiple comparisons test. **(J–L)** Cell migration ability of SW900 cells after lentiviral overexpression of STYK1 and FoxO1AAA, evaluated by RTCA and crystal violet staining; *P*′ = 0.0002, ANOVA followed by the Tukey’s multiple comparisons test. **(M–O)** Cell invasion potential after co-transduction with lentiviruses; *P*′ = 0.0210, ANOVA followed by the Tukey’s multiple comparisons test. **(P–U)** Representative immunoblots and semiquantitative analysis of the p-FoxO1/FoxO1 ratio and EMT biomarkers after co-transduction with lentiviruses; *P*′ = 0.0274, *P*′ = 0.0312, *P*′ = 0.0034, *P*′ = 0.0432 and *P*′ = 0.0252, ANOVA followed by the Tukey’s multiple comparisons test. **(V)** Typical IHC of STYK1 and p-FoxO1 protein expression in NSCLC samples (*n* = 70). **(W)** Correlation between STYK1 and p-FoxO1 expression levels in NSCLC samples; *P* = 0.0034, Spearman correlation analysis; Spearman *r* = 0.3455. LV-CON, control SW900 cells; LV-STYK1, SW900 cells overexpressing STYK1; sh-CON, control Calu-1 cells; sh-STYK1, Calu-1 cells transfected with shRNA plasmids targeting *STYK1*; LV-STYK1 + FoxO1AAA, SW900 cells with lentiviral overexpression of both STYK1 and FoxO1AAA. ADC, adenocarcinoma; SCC, squamous cell carcinoma.

To determine the role of FoxO1 in the upregulation of migration, invasion, and EMT by STYK1, the relative expression levels of p-FoxO1 and total FoxO1 were determined using western blots in SW900 and Calu-1 cells after manipulations of STYK1 expression. The ratio of p-FoxO1 to FoxO1 was measured to determine the level of FoxO1 phosphorylation, which, in turn, negatively correlates with the extent of FoxO1 activation. We found that higher levels of the p-FoxO1/FoxO1 ratio were accompanied by STYK1 upregulation, and *vice versa* (Figures 5A–F). These results indicate that STYK1 might influence cellular behaviors by phosphorylating and inactivating FoxO1.

We then transduced STYK1 overexpressing SW900 cells with lentiviruses encoding FoxO1AAA and obtained cells with constitutively active FoxO1 to further investigate the mechanism underlying the interaction between STYK1 and FoxO1. RT-qPCR and western blotting confirmed successful transduction of SW900 cells and expression of FoxO1AAA (Figures 5G–I). Following the RTCA and detection of EMT biomarkers, we observed that overexpression of FoxO1AAA reversed the positive effect of STYK1 overexpression on cell migration, invasion, and EMT (Figures 5J–U).

Furthermore, STYK1 and p-FoxO1 expression levels were evaluated using IHC of NSCLC tissue microarrays from another 70 patients with NSCLC (Figure 5V). After quantification, the Spearman correlation analysis demonstrated that STYK1 expression was positively related to p-FoxO1 expression in NSCLC samples (Figure 5W).

Therefore, our data indicate that STYK1 enhances migration, invasion, and EMT in NSCLC cells possibly by repressing FoxO1.

## Discussion

In the present study, it was demonstrated that STYK1 was upregulated in NSCLC tissues and cell lines, and that such overexpression correlated with poor prognosis of patients with NSCLC after surgery. Moreover, enhanced STYK1 expression activated the migration, invasion, and EMT of NSCLC cells, and experiments in xenografted mice confirmed that STYK1 overexpression stimulated metastasis in NSCLC. Furthermore, the stimulatory effects of STYK1 on metastatic properties were concomitant with FoxO1 phosphorylation and, consequently, with inactivation of FoxO1. Collectively, our results clarify oncogenic properties of STYK1 and shed light on new targets for better control of NSCLC.

Our results confirmed that STYK1 was overexpressed in NSCLC, indicating that STYK1 could play a significant role in NSCLC progression. This tendency is consistent with STYK1 overexpression in many other malignancies (Moriai et al., 2006; Jackson et al., 2009; Kondoh et al., 2009; Orang et al., 2014; Chen et al., 2016, 2017; Hu et al., 2018), except for gastric cancer (Fang et al., 2018) and some cases of castration-naïve prostate cancer (Chung et al., 2009). In addition, *in vitro* and *in vivo* experiments demonstrated that the upregulation of STYK1 expression caused epithelial NSCLC cells to acquire mesenchymal features, to migrate, and to invade, which suggests that STYK1 facilitates metastasis of NSCLC cells to distant sites. These results are supported by our data on survival and by prognostic analysis, indicating that high expression of STYK1 was related to advanced N stage and AJCC 8th stage, and lower postoperative 5-year survival rate in patients with NSCLC, in agreement with the results of other studies (Orang et al., 2014; Hu et al., 2015; Wang et al., 2016; Chen et al., 2017; Zhao et al., 2017; Hu et al., 2018). Altogether, STYK1 likely promotes the metastasis of NSCLC by inducing the EMT process, and targeting STYK1 may be a rational approach to tackle NSCLC progression.

We also observed that the inactivation of FoxO1 could be an essential factor whereby STYK1 promotes metastasis and EMT in NSCLC. To date, FoxO1 is considered a tumor suppressor, and its functions mainly depend on post-transcriptional modifications (Kim et al., 2018; Xing et al., 2018). Phosphorylation is the most important regulatory mechanism of FoxO1 activity. As a member of the receptor protein tyrosine kinase superfamily, STYK1 potentially can phosphorylate FoxO1, leading to the inactivation of the latter and stimulation of subsequent tumorigenesis. In the present study, we found that the level of phosphorylated FoxO1 was positively related to STYK1 expression in NSCLC tissues and cell lines. Furthermore, overexpression of the constitutively active FoxO1 reversed the positive effect of STYK1 on the migration, invasion, and EMT in NSCLC. These results not only are in line with other studies that reported inhibitory effect of FoxO1 on metastasis and EMT in malignancies (Kojima et al., 2010; Yang et al., 2014; Ko et al., 2015; Du et al., 2016; Dong et al., 2017; Gao et al., 2018), but also demonstrate another mechanism underlying oncogenic properties of STYK1.

We utilized the RTCA to study changes in cellular migration and invasion abilities. RTCA detects the impedance change caused by the cells attached to the electrode sheet in real time and reflects the number of cells that have already migrated or invaded by calculating the cell index (Solly et al., 2004). Compared with the classic Transwell migration and invasion assays, RTCA achieves better real-time and quantitative monitoring of migrated or invaded cells, thus minimizing the measurement error. Therefore, by combining the qualitative analysis of crystal violet staining and the quantitative analysis of the normalized cell index, our study likely reveals the effects of STYK1 on cell migration and invasion more objectively.

The present study had some limitations. We failed to determine STYK1 as an independent prognostic factor in the present study, and we believe that the selection bias may have contributed to this negative result. First, as we only included patients who underwent surgical treatment, our results may not be representative of all stages of NSCLC. Second, given that the completeness of the 5-year follow-up data was one of the prior criteria, we included surgical cases dating from 2011 to 2014. However, because of the lower prevalence of lung cancer screening at that time as well as due to the lower specificity of the common lung cancer symptoms, about 47% of patients included were at an advanced stage. However, considering that high STYK1 expression correlates with an advanced stage and lower 5-year survival rate in our study and STYK1 being one of the prognostic factors in other malignancies, we believe STYK1 might still be an independent prognostic factor in NSCLC, and further studies would be needed to verify this.

We found an interesting trend discrepancy between the *STYK1* mRNA and protein levels in NSCLC cell lines. We believe it is due to post-transcription regulation. Firstly, the STYK1 degradation might be reduced in tumor cell lines compared to that in HBE cells because of the UPS-SUMOylation imbalance. Our previous study showed SUMO1 protein was upregulated and was positively related to STYK1 level in NSCLC, leading to the suppression of STYK1 degradation (Ke et al., 2018). Secondly, STYK1 inhibition of non-coding RNAs might be weaker in tumor cell lines than that in HBE. Studies revealed miR-203 and miR-27, which potentially target STYK1, were downregulated in NSCLC, and such downregulation of miRNAs correlated with the aggressiveness of NSCLC cells (Jiang et al., 2014; Hu et al., 2016). However, the lower *STYK1* mRNA level in NSCLC cell lines is contradictory to the results of ONCOMINE data analyses. Therefore, we performed the supplementary RT-qPCR to test the *STYK1* mRNA level in tumor samples and corresponding normal lung tissues of some patients included in the study. We observed that *STYK1* mRNA level was upregulated in 5 squamous cell carcinomas and 5 adenocarcinomas (Supplementary Figure 1), which was consistent with ONCOMINE data. As for the trend discrepancy between *STYK1* mRNA level in tumor tissues and that in cell lines, after excluding the possibility of non-specificity of primers (Supplementary Figure 2), we hypothesize the tumor microenvironment might be one of the contributors, and further studies are needed to explore more regulatory mechanisms.

## Conclusion

This study revealed that STYK1 was upregulated in NSCLC and its high expression level correlated with poorer clinical outcomes. In addition, STYK1 could suppress FoxO1 activity, subsequently promoting metastasis and epithelial-mesenchymal transition in NSCLC. These findings contribute to better understanding of the mechanisms of STYK1-induced NSCLC progression and of the control of NSCLC metastasis.

## Data Availability Statement

The original contributions presented in the study are included in the article/Supplementary Material, further inquiries can be directed to the corresponding authors.

## Ethics Statement

The studies involving human participants were reviewed and approved by the Institutional Ethics Review Board of Tangdu Hospital. The patients/participants provided their written informed consent to participate in this study. The animal study was reviewed and approved by the Laboratory Animal Welfare and Ethics Committee of the Air Force Medical University.

## Author Contributions

XL, ZZ, and JZo designed the study. YL, FL, and XW performed the experiments. JZn, JX, YS, and MW supervised the project. YL and FL analyzed the data. YL drafted the manuscript, which was corrected by XL, ZZ, and JZo. All authors contributed to completing this study, read, and approved the final manuscript.

## Conflict of Interest

The authors declare that the research was conducted in the absence of any commercial or financial relationships that could be construed as a potential conflict of interest.

## Footnotes

1 www.oncomine.org
