# Supplementary material for: STYK1/NOK Promotes Metastasis and Epithelial-Mesenchymal Transition in Non-small Cell Lung Cancer by Suppressing FoxO1 Signaling
Source: Front Cell Dev Biol. 2021 Jul 6;9:621147. doi: 10.3389/fcell.2021.621147 (PMC8290174; doi:10.3389/fcell.2021.621147)
Supplement: Supplementary file 1 [file Data_Sheet_1.DOCX]

Supplementary Material

## Supplementary Figures

**
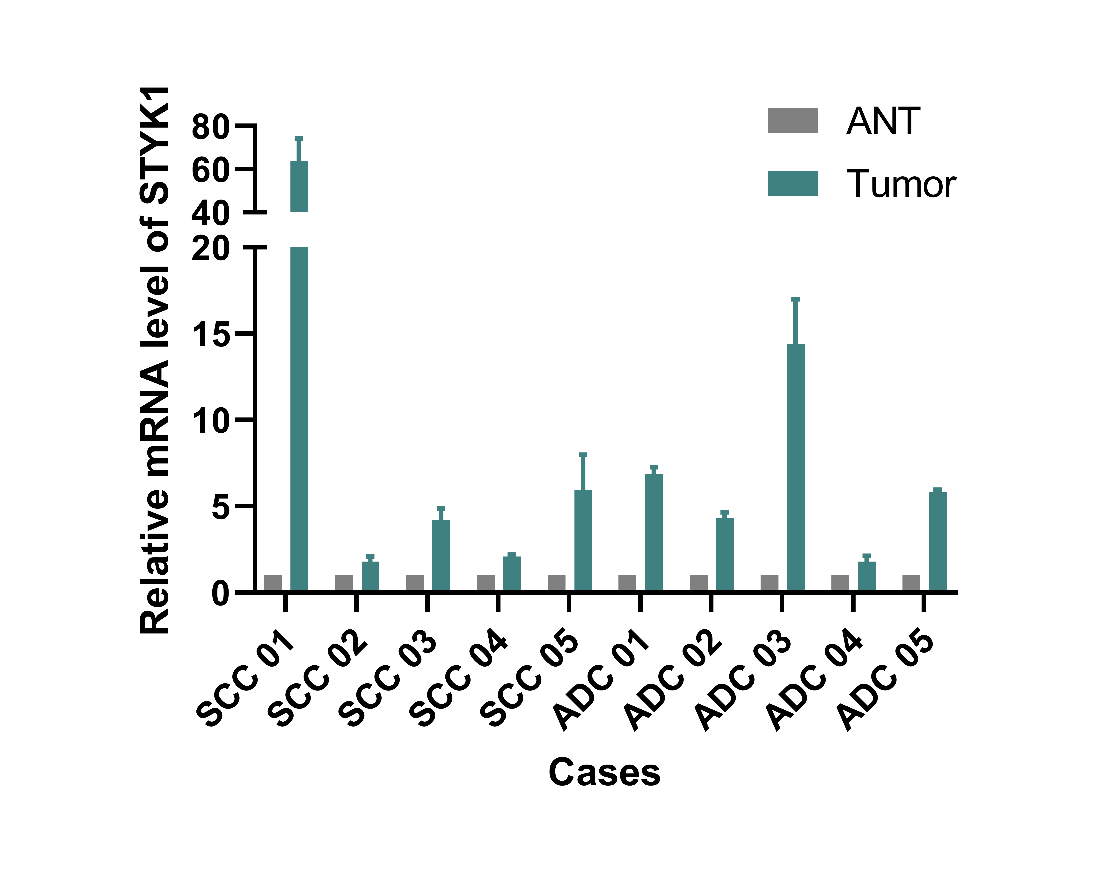
**

**Supplementary Figure 1. *STYK1* mRNA is upregulated in 10 NSCLC tissues (5 squamous cell carcinomas, 5 adenocarcinomas).** The RT-qPCR was performed using the same reaction conditions as described in the manuscript; all the reactions were replicated 3 times. Reagents: RNAprep Pure Tissue Kit (Cat.: DP431; TIANGEN BIOTECH, Beijing, China); Thermo Scientific RevertAid First Strand cDNA Synthesis Kit (Cat.: K1622; Thermo Fisher Scientific, Waltham, MA, USA); UltraSYBR Mixture (Cat.: CW0957; CWBIO, Beijing, China). Profile of 40 thermal cycles: denaturation for 40 s at 95 °C, annealing for 30 s at 61 °C, and extension for 45 s at 72 °C. Primer sequences: STYK1, forward (5’-TCGAGCCAATATGAACACTGGG-3’) and reverse (5’-TCGCCCTAAGAAATCTTGTACCT-3’); β-actin, forward (5’-CTCCATCCTGGCCTCGCTGT-3’) and reverse (5’-GCTGTCACCTTCACCGTTCC-3’). The results were normalized to the level of β-actin mRNA; with the corresponding adjacent normal lung tissue as the calibrator, the relative mRNA level of *STYK1* in each NSCLC tissue was quantified by the 2^−ΔΔCt^ method. ANT, adjacent normal lung tissue; SCC, squamous cell carcinoma; ADC, adenocarcinoma.

**
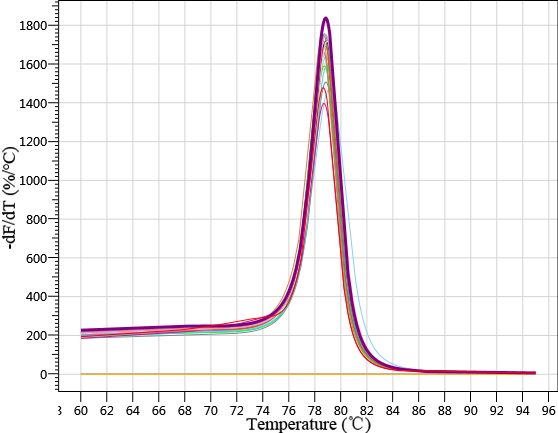
**

**Supplementary Figure 2. Melt curves from RT-qPCR of STYK1 gene.** An amplicon from STYK1 revealed a single peak following melt curve analysis.
